# Catarrh

**Published:** 1874-07

**Authors:** 


### CATARRH.

This disease is becoming so prevalent and is fruitful of so many serious results, that charlatans have taken advantage of this circumstance to flood the country with printed announcements of nostrums for its cure, and traveling doctors have humbugged the people into spending fabulous sums of money for periodical spurts of treatment, which has been of no value, or at best, of temporary benefit.

The truth is, that while catarrh is curable under the care of resident specialists, yet the same causes always exist to reproduce the disease; and when the attack again occurs, our traveling humbug, even if he has succeeded in temporarily relieving the patient, has decamped with his prepaid fee, and leaves the patient in worse plight than before.

Catarrh, to be cured, requires careful and skillful treatment by means of the various excellent appliances, recently presented to the profession, by scientific specialists, who make diseases of the respiratory organs their special study. When the disease is cured, the patient is required to call upon the physician every fall and spring, (when renewed attacks usually occur) when, with very little medication, catarrh is prevented. In this manner, the worst forms of catarrh are curable, and the patient relieved of all the distressing symptoms incurred through its ravages.

In this climate, we have to deal with much deafness, the result of catarrh. This will not appear so strange to the general reader when they are informed that the same mucus lining which is distributed over the throat and nostrils, extends up, through the *eustachian tubes,* into the middle ear, just behind the drum.

Persons who have taken a bad " cold in the head," are conscious of this communication; for then the tubes become obstructed, either from congestion or accumulation of mucus, giving the ears a "stopped up" feeling, when the hearing becomes very dull. In catarrhal deafness, in addition to this sensation, there exists noises of various kinds in the ears, most distressing to endure. Chirping of crickets, puffing of steam, roar of falling water, tingling of bells, singing of the tea-kettle, etc., etc., are the sounds usually described by the patient.

Sometimes this is accompanied with pain in the head, usually between the eyes, and immediately behind the ears. Thumping the head with the fingers, behind the ears, is painful, and often the entire scalp is sensitive to the touch. The discharges from nose and throat vary in different individuals. In many the expectoration is profuse, a constant dropping of tough tenacious mucus escaping from the nostrils into the throat, requiring much "hawking" to dislodge it. In others the secretions are dry, the patient rarely discovering that any are present, until later in the attack when it takes on the form of what is known to the physician as *ozanœ*. In this stage, the secretions are so dry as to form hard, dry and green looking scabs in the throat and nose. When they are removed by means of snuffing warm water up the nostrils, they come out in almost perfect casts of the parts diseased. These discharges are horridly offensive to sight and smell, and are indicative of the last stages of a very dangerous catarrh.

All these symptoms are nearly always curable, in the hands of the proper physicians, which by no means are those who stop for a few days in the hotels of our numerous cities and villages.

